# Urban Microbiomes in Narita, Chiba, Japan: Shotgun Metagenome Sequences of a Train Station

**DOI:** 10.1128/mra.01092-22

**Published:** 2022-12-14

**Authors:** Dewa A. P. Rasmika Dewi, Yuh Shiwa, Krista Ryon, Christopher E. Mason, Tetsuya Matsumoto, Haruo Suzuki

**Affiliations:** a Department of Infectious Disease, School of Medicine, International University of Health and Welfare, Narita, Chiba, Japan; b Faculty of Medicine and Health Sciences, Udayana University, Bali, Indonesia; c Department of Molecular Microbiology, Tokyo University of Agriculture, Tokyo, Japan; d NODAI Genome Research Center, Tokyo University of Agriculture, Tokyo, Japan; e Department of Physiology and Biophysics, Weill Cornell Medicine, New York, New York, USA; f The HRH Prince Alwaleed Bin Talal Bin Abdulaziz Alsaud Institute for Computational Biomedicine, Weill Cornell Medicine, New York, New York, USA; g The WorldQuant Initiative for Quantitative Prediction, Weill Cornell Medicine, New York, New York, USA; h Graduate School of Medicine, International University of Health and Welfare, Narita, Chiba, Japan; i Institute for Advanced Biosciences, Keio University, Tsuruoka, Yamagata, Japan; j Faculty of Environment and Information Studies, Keio University, Fujisawa, Kanagawa, Japan; Montana State University

## Abstract

Here, we performed shotgun metagenome sequencing of swab samples collected on floors at a train station in Narita City, Chiba, Japan. The taxonomic analysis revealed that *Actinobacteria* and *Proteobacteria* were the dominant phyla. The data will contribute to insight into the microbiome community on the surfaces of urban built environments.

## ANNOUNCEMENT

Increasing urbanization creates a high-density urban environment and leads to a continual interaction between humans and urban microorganisms (1, 2) that influence human health and biosafety (2, 3). Urban transit systems, such as trains and buses, host large numbers of passengers (4) and may facilitate constant contact of human commensals with environmental microbes (1). This system offers an ideal model to examine transfers of the urban and interindividual community microbiome (1, 4), and floors have the highest diversity of characterized microbiomes compared with other surfaces (5). In this study, we used shotgun metagenomic sequencing to profile microbial communities on floors in a train station.

Two floor samples were collected at the public area in a train station in Narita City, Chiba Prefecture, Japan, in 2021 using Isohelix swabs (Cell Projects Ltd., Maidstone, UK) prefilled with 400 mL of DNA/RNA Shield medium in barcoded tubes (Zymo Research Co., CA). Sampling supplies were provided by the MetaSUB International Consortium (http://metasub.org). Metagenomic DNA was extracted using the ZymoBiomics DNA miniprep kit (Zymo Research Co.) according to the manufacturer’s instructions. DNA quantitation was done using Qubit double-stranded DNA (dsDNA) high-sensitivity (HS) assay kits (ThermoFisher Scientific Inc., MA) according to the kit procedure. DNA was then subjected to shotgun metagenomic sequencing using the 2 × 150-bp paired-end read DNBSEQ-G400RS high-throughput sequencing set (MGITech Co., Tokyo, Japan) (6) performed at Genome Lead Co. Ltd. (Kagawa, Japan). The metagenomic library was prepared using the MGIEasy FS DNA library prep set (MGITech Co.) with 10 cycles of real-time PCR amplification.

Initially, 1,515,930 sequence reads were generated with a mean length of 150 bp. Quality trimming of sequence reads was then performed by applying the program Fastp 0.23.2 (7) and yielded 1,455,502 paired-end reads with an average read length of 148 bp. The reads were mapped to a database of clade-specific marker genes using Bowtie 2 with the “--very-sensitive” option (8), and the relative abundance of microbial taxa in the metagenome was estimated using the coverage of clade-specific marker genes using MetaPhlAn 3 (9) and was visualized using the Pavian package (10) running on the R program (https://www.r-project.org/).

**FIG 1:**
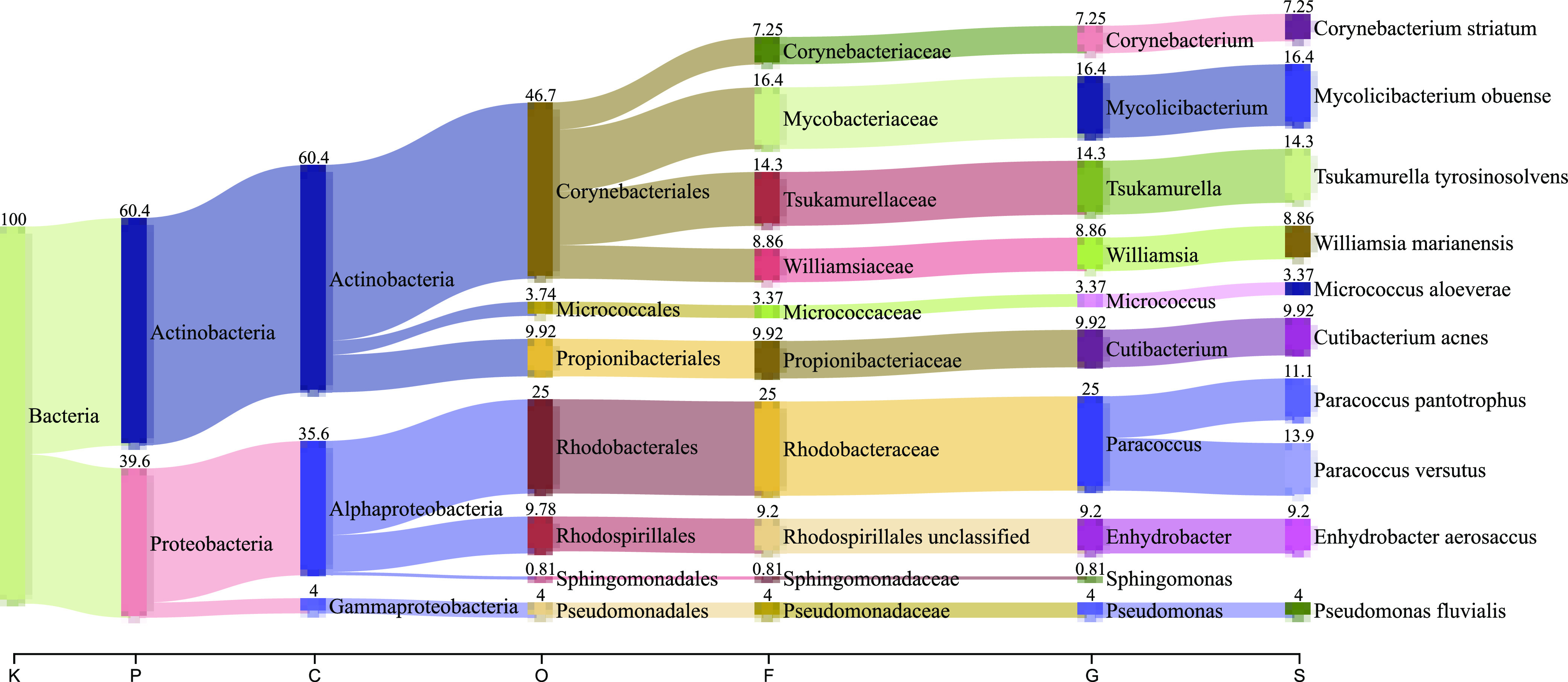
Taxonomic classification of microbial communities recovered from metagenomic shotgun sequence using MetaPhlAn 3.0 and visualized with Pavian package. Taxonomic ranks are abbreviated as follows: K, kingdom; P, phylum; C, class; O, order; F, family; G, genus; and S, species.

Taxonomic analysis was shown in Fig. 1; all reads were assigned to *Bacteria* which consisted of phyla *Actinobacteria* (60.4%) and *Proteobacteria* (39.6%), of which both are commonly identified in the built environment (2, 11). Within *Actinobacteria*, *Corynebacteriales* (46.7%) and *Mycobacteriaceae* (16.4%) represented the most abundant order and family, respectively. The most prominent genus was *Mycolicibacterium* which was dominated by Mycolicibacterium obuense. The following other species were also identified: Tsukamurella tyrosinosolvens (14.3%), Cutibacterium acnes (9.9%), Williamsia marianensis (8.9%), Corynebacterium striatum (7.3%), and Micrococcus aloeverae (3.4%). *C. acnes*, a commensal human skin flora, has been reported as the most relatively abundant microbial species in metagenomic samples collected in 60 cities (2). The phylum *Proteobacteria* comprises the class *Alphaproteobacteria* (35.6%) and *Gammaproteobacteria* (4%). Within the *Alphaproteobacteria*, the most abundant genus was *Paracoccus* (25%) belonging to the family *Rhodobacteraceae* and the order *Rhodobacterales*. This genus comprised the species Paracoccus versutus (13.9%) and Paracoccus pantotrophus (11.1%). Pseudomonas fluvialis (4%) was identified within the class *Gammaproteobacteria*. This information contributes to catalogizing the unique and complex microbial communities in urban public transit systems.

### Data availability.

The raw sequencing reads have been deposited in the DDBJ Sequence Read Archive (DRA) under the accession number DRR403243. The sample information is available under the DDBJ BioSample accession number SAMD00520086. The project information is available under the DDBJ BioProject accession number PRJDB14136, under the umbrella BioProject accession number PRJDB13760. The code and scripts to run the bioinformatics tools are available online at https://github.com/haruosuz/bioproject. Default parameters were used for all software unless otherwise noted.

## References

[1] Afshinnekoo E, Meydan C, Chowdhury S, Jaroudi D, Boyer C, Bernstein N, Maritz JM, Reeves D, Gandara J, Chhangawala S, Ahsanuddin S, Simmons A, Nessel T, Sundaresh B, Pereira E, Jorgensen E, Kolokotronis S-O, Kirchberger N, Garcia I, Gandara D, Dhanraj S, Nawrin T, Saletore Y, Alexander N, Vijay P, Hénaff EM, Zumbo P, Walsh M, O'Mullan GD, Tighe S, Dudley JT, Dunaif A, Ennis S, O'Halloran E, Magalhaes TR, Boone B, Jones AL, Muth TR, Paolantonio KS, Alter E, Schadt EE, Garbarino J, Prill RJ, Carlton JM, Levy S, Mason CE. 2015. Geospatial resolution of human and bacterial diversity with city-scale metagenomics. Cell Syst 1:72–87. doi:10.1016/j.cels.2015.01.001. 26594662 PMC4651444

[2] Danko D, Bezdan D, Afshin EE, Ahsanuddin S, Bhattacharya C, Butler DJ, Chng KR, Donnellan D, Hecht J, Jackson K, Kuchin K, Karasikov M, Lyons A, Mak L, Meleshko D, Mustafa H, Mutai B, Neches RY, Ng A, Nikolayeva O, Nikolayeva T, Png E, Ryon KA, Sanchez JL, Shaaban H, Sierra MA, Thomas D, Young B, Abudayyeh OO, Alicea J, Bhattacharyya M, Blekhman R, Castro-Nallar E, Cañas AM, Chatziefthimiou AD, Crawford RW, De Filippis F, Deng Y, Desnues C, Dias-Neto E, Dybwad M, Elhaik E, Ercolini D, Frolova A, Gankin D, Gootenberg JS, Graf AB, Green DC, Hajirasouliha I, Hastings JJA, The International MetaSUB Consortium., et al. 2021. A global metagenomic map of urban microbiomes and antimicrobial resistance. Cell 184:3376–3393.e17. doi:10.1016/j.cell.2021.05.002. 34043940 PMC8238498

[3] Nicolaou N, Siddique N, Custovic A. 2005. Allergic disease in urban and rural populations: increasing prevalence with increasing urbanization. Allergy 60:1357–1360. doi:10.1111/j.1398-9995.2005.00961.x. 16197466

[4] Hsu T, Joice R, Vallarino J, Abu-Ali G, Hartmann EM, Shafquat A, DuLong C, Baranowski C, Gevers D, Green JL, Morgan XC, Spengler JD, Huttenhower C. 2016. Urban transit system microbial communities differ by surface type and interaction with humans and the environment. mSystems 1:e00018-16. doi:10.1128/mSystems.00018-16. 27822528 PMC5069760

[5] Klimenko NS, Tyakht AV, Toshchakov SV, Shevchenko MA, Korzhenkov AA, Afshinnekoo E, Mason CE, Alexeev DG. 2020. Co-occurrence patterns of bacteria within microbiome of Moscow subway. Comput Struct Biotechnol J 18:314–322. doi:10.1016/j.csbj.2020.01.007. 32071708 PMC7016200

[6] Jia Y, Zhao S, Guo W, Peng L, Zhao F, Wang L, Fan G, Zhu Y, Xu D, Liu G, Wang R, Fang X, Zhang H, Kristiansen K, Zhang W, Chen J. 2022. Sequencing introduced false positive rare taxa lead to biased microbial community diversity, assembly, and interaction interpretation in amplicon studies. Environ Microbiome 17:43. doi:10.1186/s40793-022-00436-y. 35978448 PMC9387074

[7] Chen S, Zhou Y, Chen Y, Gu J. 2018. Fastp: an ultra-fast all-in-one FASTQ preprocessor. Bioinformatics 34:i884–i890. doi:10.1093/bioinformatics/bty560. 30423086 PMC6129281

[8] Langmead B, Salzberg SL. 2012. Fast gapped-read alignment with Bowtie 2. Nat Methods 9:357–359. doi:10.1038/nmeth.1923. 22388286 PMC3322381

[9] Beghini F, McIver LJ, Blanco-Míguez A, Dubois L, Asnicar F, Maharjan S, Mailyan A, Manghi P, Scholz M, Thomas AM, Valles-Colomer M, Weingart G, Zhang Y, Zolfo M, Huttenhower C, Franzosa EA, Segata N. 2021. Integrating taxonomic, functional, and strain-level profiling of diverse microbial communities with bioBakery 3. Elife 10:e65088. doi:10.7554/eLife.65088. 33944776 PMC8096432

[10] Breitwieser FP, Salzberg SL. 2020. Pavian: interactive analysis of metagenomics data for microbiome studies and pathogen identification. Bioinformatics 36:1303–1304. doi:10.1093/bioinformatics/btz715. 31553437 PMC8215911

[11] Merino N, Zhang S, Tomita M, Suzuki H. 2019. Comparative genomics of bacteria commonly identified in the built environment. BMC Genomics 20:92. doi:10.1186/s12864-018-5389-z. 30691394 PMC6350394

